# Supplementary material for: Efficacy, Immunogenicity, and Safety of Pertussis Vaccine During Pregnancy: A Meta-Analysis
Source: Vaccines (Basel). 2025 Jun 20;13(7):666. doi: 10.3390/vaccines13070666 (PMC12298825; doi:10.3390/vaccines13070666)
Supplement: Supplementary file 1 [file vaccines-13-00666-s001.zip › Supplemental.pdf]

Supplemental Table S1 Search strategy

#1AND#2AND#3AND#4
Search terms for PubMed (n =808), until 16 February 2025
#1(((((Pertussis) OR (whooping cough)) OR (DPT)) OR (DaPT)) OR (Diphtheria-Tetanus-acellular Pertussis)) OR (Diphtheria-Tetanus-Pertussis) (38859)
#2(((((pregnan*) OR (gestation)) OR (pre-partum)) OR (maternal)) OR (Maternal-Fetal)) OR (Transplacental) (891065)
#3((((((((((((((((effectiveness)) OR (efficacy)) OR (Incidence)) OR (morbidity)) OR (prevalence)) OR (mortality)) OR (hospitaliz*)) OR (antibody)) OR (antibodies)) OR (immunogenicity)) OR (immunity)) OR (immune response)) OR (IgG)) OR (safety)) OR (AEFI)) OR (adverse effect)) OR (side effect) (6252474)
#4((Infant) OR (infants)) OR (newborn) (589751)
#1AND#2AND#3AND#4
Search terms for Embase (n =1696), until 16 February 2025
#1‘pertussis’/exp OR ‘pertussis’ OR ‘whooping cough’/exp OR ‘whooping cough’ OR ‘dpt’/exp OR ‘dpt’ OR ‘dapt’/exp OR ‘dapt’ OR ‘diphtheria-tetanus-acellular pertussis’ OR ‘diphtheria-tetanus-pertussis’ (72093)
#2‘pregnan*’ OR ‘gestation’ OR ‘pre-partum’ OR ‘maternal’ OR ‘maternal-fetal’ OR ‘transplacental’ (1610856)
#3‘effectiveness’ OR ‘efficacy’ OR ‘incidence’ OR ‘morbidity’ OR ‘prevalence’ OR ‘mortality’ OR ‘hospitaliz*’ OR ‘antibody’ OR ‘antibodies’ OR ‘immunogenicity’ OR ‘immunity’ OR ‘immune response’ OR ‘igg’ OR ‘safety’ OR ‘aefi’ OR ‘adverse effect’ (11184781)
#4‘infant’ OR ‘infants’ OR ‘newborn’ (1656865)
Search terms for Web of Science (n =2104), until 16 February 2025
#1 'Pertussis' (Topic) or 'whooping cough' (Topic) or 'DPT' (Topic) or 'DaPT' (Topic) or 'Diphtheria-Tetanus-acellular Pertussis' (Topic) or 'Diphtheria-Tetanus-Pertussis' (Topic) and Preprint Citation Index (Exclude – Database) (65394)
#2 'pregnan*' (Topic) or 'gestation' (Topic) or 'pre-partum' (Topic) or 'maternal' (Topic) or 'Maternal-Fetal' (Topic) or 'Transplacental' (Topic) and Preprint Citation Index (Exclude – Database) (1864348)
#3 'effectiveness' (Topic) or 'efficacy' (Topic) or 'Incidence' (Topic) or 'morbidity' (Topic) or 'prevalence' (Topic) or 'mortality' (Topic) or 'hospitaliz*' (Topic) or 'antibody' (Topic) or 'antibodies' (Topic) or 'immunogenicity' (Topic) or 'immunity' (Topic) or 'immune response' (Topic) or 'IgG' (Topic) or 'safety' (Topic) or 'AEFI' (Topic) or 'adverse effect' (Topic) or 'side effect' (Topic) and Preprint Citation Index (Exclude – Database) (17605011)
#4 'Infant' (Topic) or 'infants' (Topic) or 'newborn' (Topic) and Preprint Citation Index (Exclude – Database) (2039596)

Supplemental Figure S1 Study quality assessment of included randomized

controlled studies in the meta-analysis

	Risk of bias domains					
Study ID	D1	D2	D3	D4	D5	Overall
Barug D 2019	+	+	+	+	+	+
Haidara FC 2024	+	+	+	+	+	+
Hoang HT 2015	?	+	?	+	+	!
Munoz FM 2014	+	+	+	+	+	+
Halperin SA 2018	+	+	+	+	+	+
Perrett KP 2019	+	+	+	+	+	+
Villarreal Pérez JZ 2016	?	+	+	+	+	!

Domains:

D1: Randomization process

D2: Deviations from intended interventions

D3: Missing outcome data

D4: Measurement of the outcome

D5: Selection of the reported result

Judgement

+ Low risk

? Some concerns

Supplemental Table S2 Study quality assessment of included case - control studies in the meta-analysis

Studies	Selection (maximum ****)				Comparability (maximum **)		Outcome (maximum ***)			Total scores (maximum 9)
	1	2	3	4	5	6	7	8	9	
Cheuvart B et al. (2023)	*	*	*	*	*	*	*	*	*	9
López EJ et al.(2023)	*		*	*	*		*	*	*	7
Quinn HE et al. (2022)	*	*	*	*	*		*	*	*	8
Merdrignac L et al. (2022)	*		*	*	*		*	*	*	7
Godoy P et al. (2021)	*	*	*	*	*		*	*	*	8
Romanin V et al. (2019)	*	*	*	*	*	*	*	*	*	8
Fernandes EG et al. (2019)	*	*	*	*	*		*	*		7
Dabrera G et al. (2014)	*	*	*	*	*		*	*	*	8

Saul N et al. (2018)	*		*	*	*	*	*	*	*	7
Blasco JB et al. (2017)	*		*	*	*	*	*	*	*	7

For case-control studies:
1 indicates cases with independent validation;
2 consecutive or obviously representative cases;
3 community controls;
4 control group have no history of pertussis
5 study controls for the most important factor;
6 study controls for any additional factor (These criteria could be modified to indicate specific control for a second important factor);
7 ascertainments of exposure by blinded interview or record;
8 the same method of ascertainment for cases and controls;
9 the same non-response rate for both groups.

Supplemental Table S3 Subgroup analysis of anti-PT antibody levels in cord blood

Subgroup	Included studies	Pooled SMD (95 % CI)	*P*	*P* for interaction	Heterogeneity		
					Q	I^2(%)	*P*
Total	8	1.57(1.25-1.89)	<0.001		45.58	84.6	<0.001
Major vaccine manufacturers	4			0.036			
GSK	4	1.86(1.56-2.17)	<0.001		10.03	70.1	0.018
SP		1.23(0.89-1.57)	<0.001		8.96	66.5	0030
Control group intervention				0.414			
TT/Td	3	1.41(1.18-1.65)	<0.001		2.83	29.3	0.243
placebo/--	5	1.70(1.18-2.21)	<0.001		39.04	89.8	<0.001
Mean gestational weeks of vaccination				0.489			
<30 weeks	2	1.37(0.91-1.82)	<0.001		2.77	63.9	0.096
≥30 weeks	6	1.65(1.24-2.05)	<0.001		40.87	87.8	<0.001

Note: SMD, Standardized Mean Difference; CI, confidence interval; GSK, GlaxoSmithKline; SP, Sanofi Pasteur; TT, tetanus toxoid vaccine; Td, tetanus-diphtheria vaccine;

Supplemental Table S4 Subgroup analysis of anti-PRN antibody levels in cord blood

Subgroup	Included studies	Pooled SMD (95 % CI)	*P*	*P* for interaction	Heterogeneity		
					Q	I^2(%)	*P*
Total	8	2.15(1.82-2.48)	<0.001		41.32	83.1	<0.001

Major vaccine manufacturers	4			0.077			
GSK	4	2.44(2.04-2.85)	<0.001		14.98	80.0	0.002
SP		1.83(1.44-2.21)	<0.001		9.28	67.7	0.026
Control group intervention				0.952			
TT/Td	3	2.15(1.17-3.17)	<0.001		36.26	94.5	<0.001
placebo/--	5	2.18(2.02-2.34)	<0.001		2.79	0.0	0.594
Mean gestational weeks of vaccination				0.269			
<30 weeks	2	2.50(1.33-3.68)	<0.001		12.98	92.3	<0.001
≥30 weeks	6	2.04(1.74-2.34)	<0.001		19.28	74.1	0.002

Note: SMD, Standardized Mean Difference; CI, confidence interval; GSK, GlaxoSmithKline; SP, Sanofi Pasteur; TT, tetanus toxoid vaccine; Td, tetanus-diphtheria vaccine;

Supplemental Table S5 Subgroup analysis of anti-FHA antibody levels in cord blood

Subgroup	Included studies	Pooled SMD (95 % CI)	*P*	*P* for interaction	Heterogeneity		
					Q	I^2(%)	*P*
Total	7	2.25(1.81-2.68)	<0.001		50.74	88.2	<0.001
Major vaccine manufacturers				0.058			
GSK	4	2.59(2.27-2.91)	<0.001		8.60	65.1	<0.001
SP	3	1.75(0.98-2.51)	<0.001		16.30	87.7	<0.001
Control group intervention				0.329			
TT/Td	3	1.97(1.122.82)	<0.001		30.17	93.4	<0.001
placebo/--	4	2.49(2.10-2.87)	<0.001		9.52	68.5	0.023
Mean gestational weeks of vaccination				0.367			
<30 weeks	2	1.88(0.28-3.49)	<0.001		29.26	96.6	<0.001
≥30 weeks	5	2.40(2.07-2.73)	<0.001		13.79	71.0	<0.001

Note: SMD, Standardized Mean Difference; CI, confidence interval; GSK, GlaxoSmithKline; SP, Sanofi Pasteur; TT, tetanus toxoid vaccine; Td, tetanus-diphtheria vaccine;

Supplemental Table S6 Subgroup analysis of anti-PT antibody levels before primary immunization

Subgroup	Included studies	Pooled SMD (95 % CI)	*P*	*P* for interaction	Heterogeneity

					Q	I^2(%)	*P*
Total	6	1.30(0.87-1.72)	<0.001		34.87	85.7	<0.001
Major vaccine manufacturers				0.184			
GSK	2	1.72(0.92-2.53)	<0.001		7.55	86.7	0.006
SP	4	1.07(0.62-1.52)	<0.001		15.27	80.4	0.002
Control group intervention				0.984			
TT/Td	3	1.30(1.10-1.50)	<0.001		0.06	0.0	0.972
placebo/--	3	1.32(0.23-2.42)	<0.001		32.16	93.8	<0.001
Mean gestational weeks of vaccination				0.987			
<30 weeks	2	1.30(1.02-1.59)	<0.001		0.06	0.0	0.813
≥30 weeks	4	1.31(0.65-1.97)	<0.001		34.02	91.2	<0.001

Note: SMD, Standardized Mean Difference; CI, confidence interval; GSK, GlaxoSmithKline; SP, Sanofi Pasteur; TT, tetanus toxoid vaccine; Td, tetanus-diphtheria vaccine;

Supplemental Table S7 Subgroup analysis of anti-FHA antibody levels before primary immunization

Subgroup	Included studies	Pooled SMD (95 % CI)	*P*	*P* for interaction	Heterogeneity		
					Q	I^2(%)	*P*
Total	5	2.21(1.81-2.61)	<0.001		14.05	71.5	<0.001
Major vaccine manufacturers				0.176			
GSK	2	2.53(2.21-2.85)	<0.001		0.21	0.0	0.006
SP	3	1.96(1.46-2.47)	<0.001		5.66	64.6	0.002
Control group intervention				0.223			
TT/Td	3	2.01(1.53-2.49)	<0.001		8.65	76.9	0.972
placebo/--	2	2.63(2.19-3.08)	<0.001		0.0	0.0	<0.001
Mean gestational weeks of vaccination				0.495			
<30 weeks	2	2.00(1.05-2.94)	<0.001		8.54	88.3	0.813
≥30 weeks	3	2.35(1.86-2.83)	<0.001		5.15	61.2	<0.001

Note: SMD, Standardized Mean Difference; CI, confidence interval; GSK, GlaxoSmithKline; SP, Sanofi Pasteur; TT, tetanus toxoid vaccine; Td, tetanus-diphtheria vaccine;

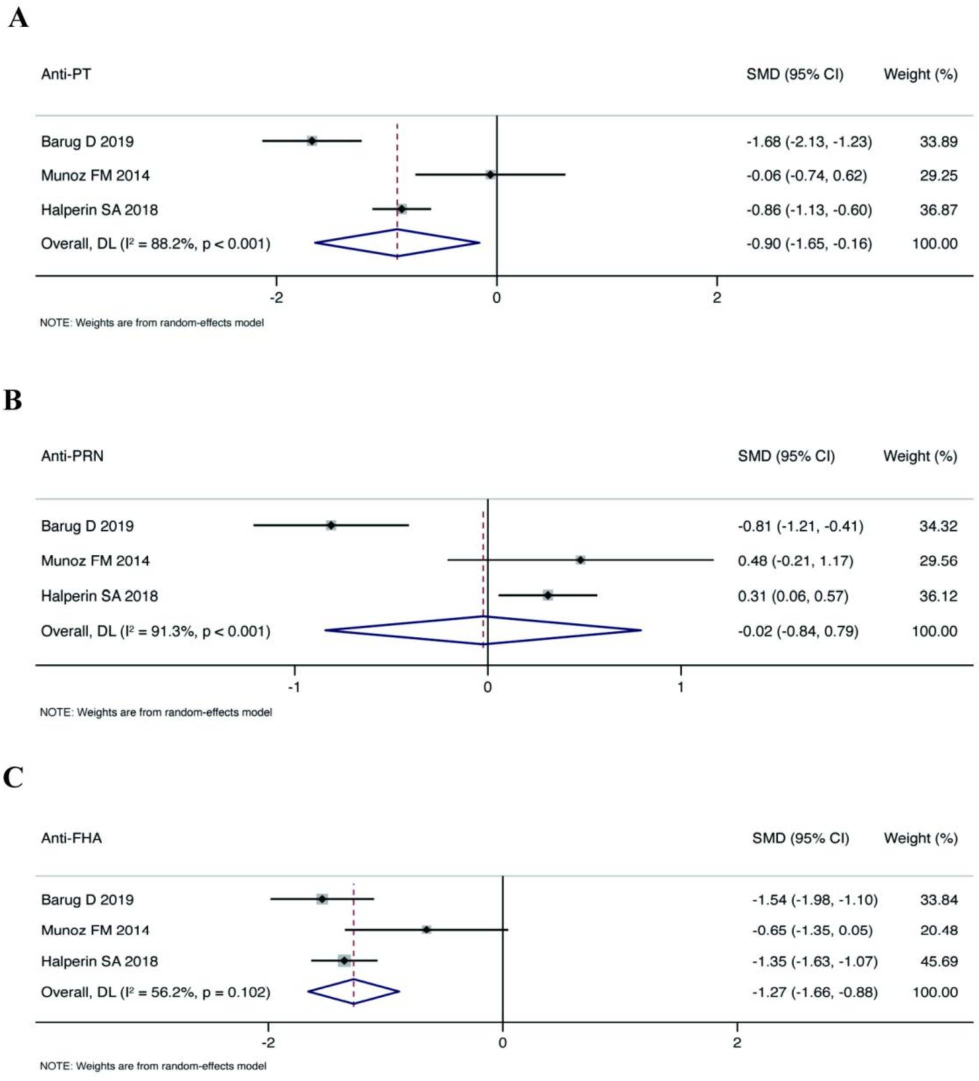

Supplemental Figure S2 Forest plots of GMCs for pertussis antibodies one-year-old infants

Note: (A) Anti-PT; (B) Anti-PRN; (C) Anti-FHA

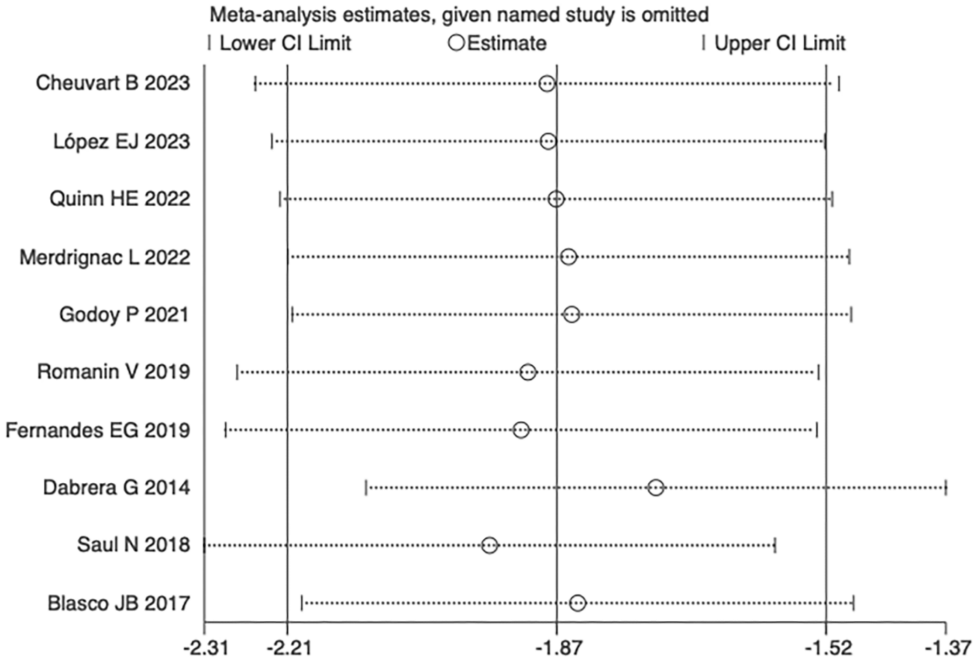

Supplemental Figure S3 Sensitivity analysis of vaccine effectiveness.

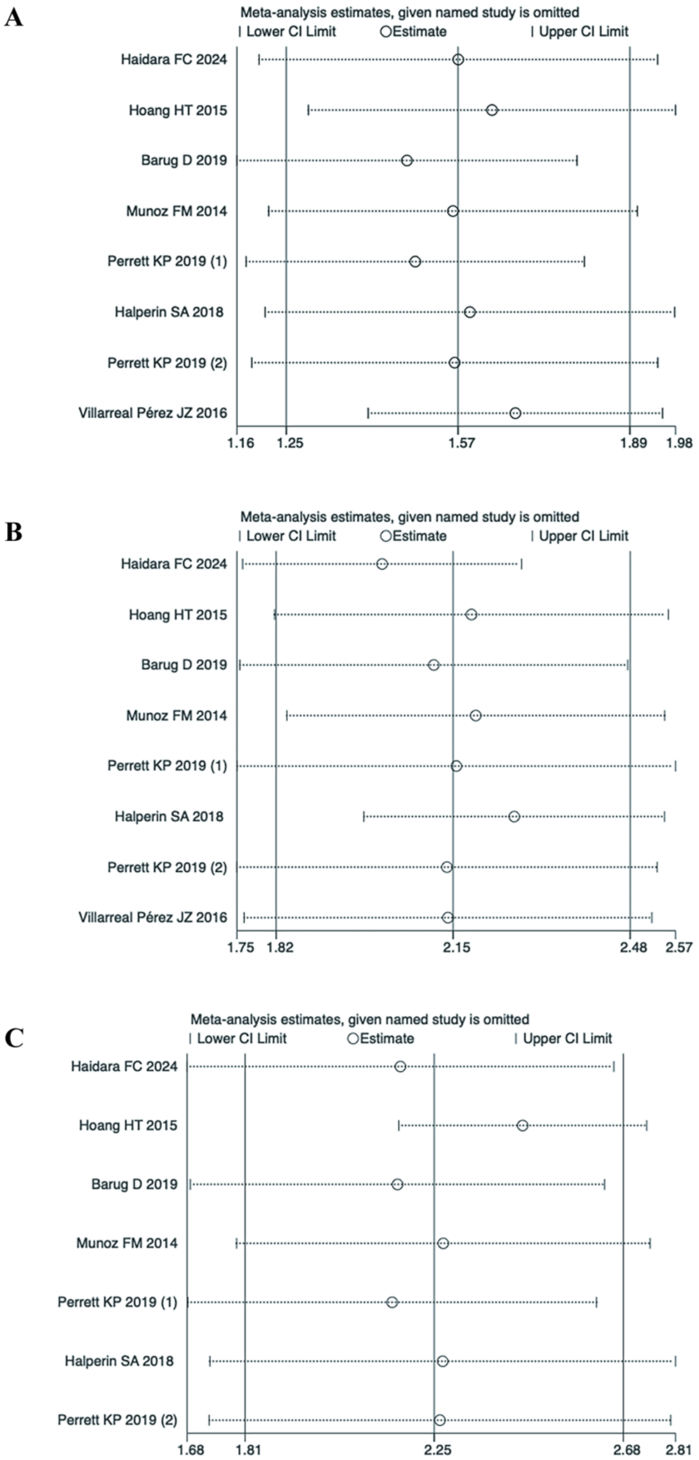

Supplemental Figure S4 Sensitivity analysis of immunogenicity results in cord blood

Note: (A) Anti-PT; (B) Anti-PRN; (C) Anti-FHA

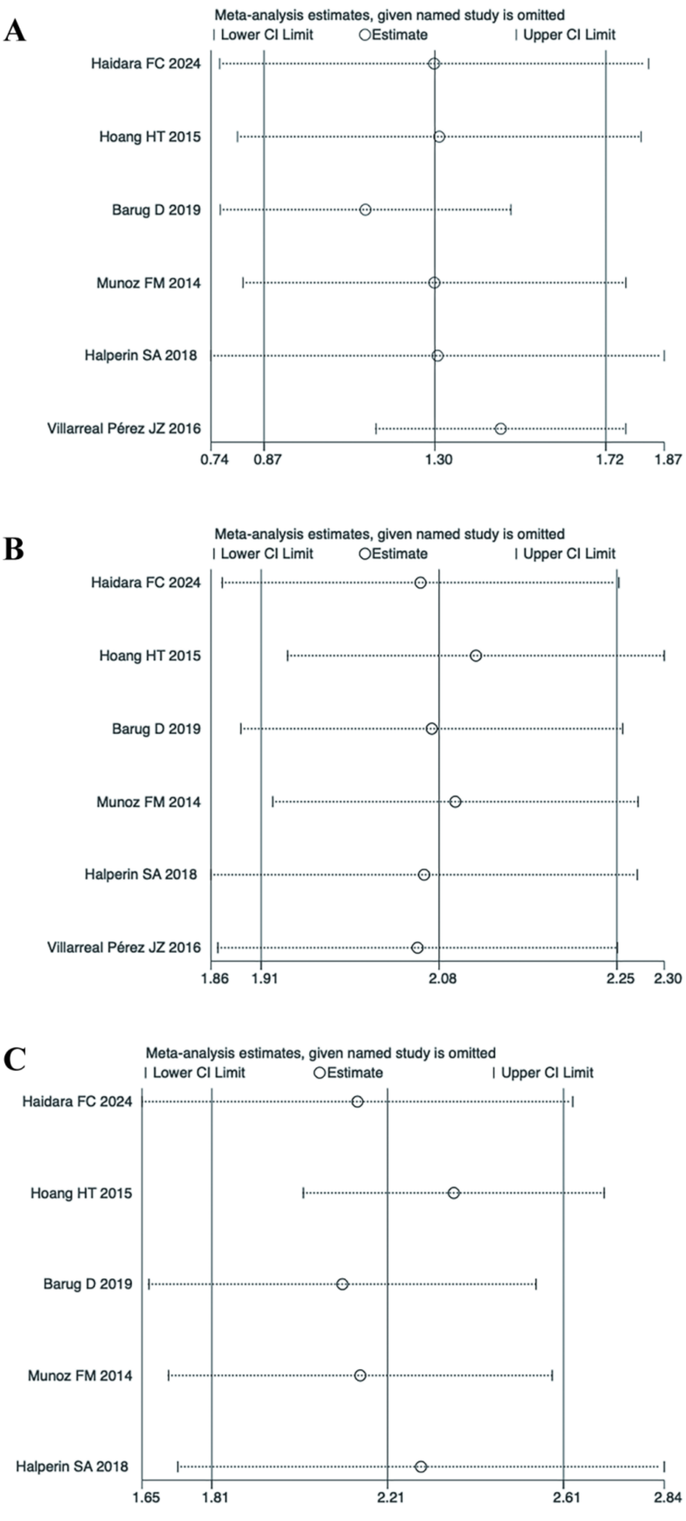

Supplemental Figure S5 Sensitivity analysis of antibody level before infant primary immunization.

Note: (A) Anti-PT; (B) Anti-PRN; (C) Anti-FHA

A

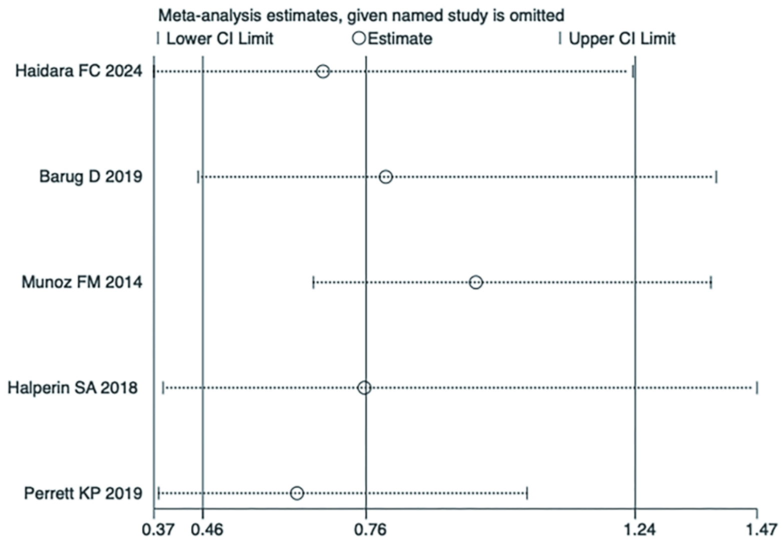

B

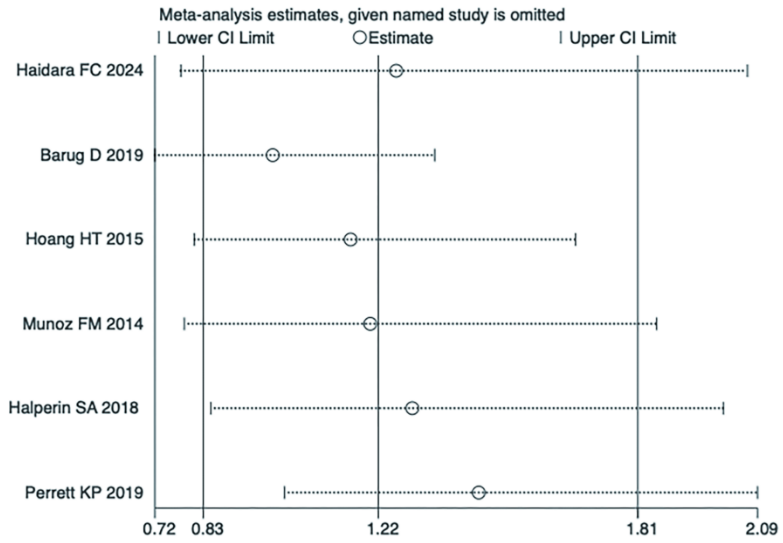

Supplemental Figure S6 Sensitivity analysis of safety results.

Note: (A) infants; (B) pregnant woman

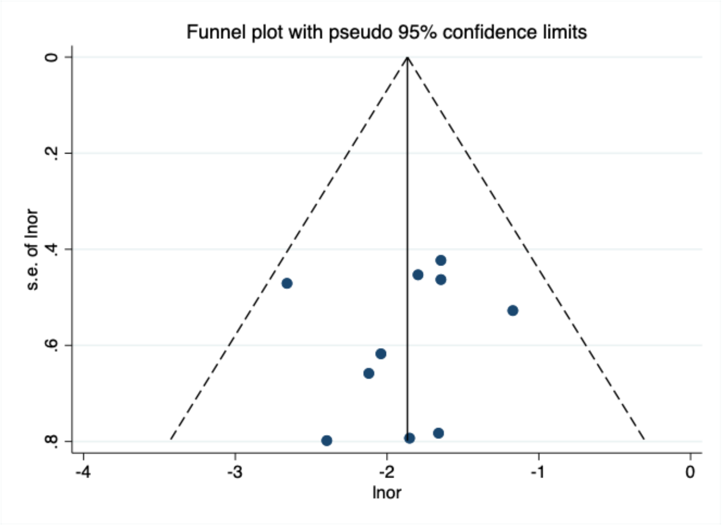

Supplemental Figure S7 Funnel plot on vaccine effectiveness studies

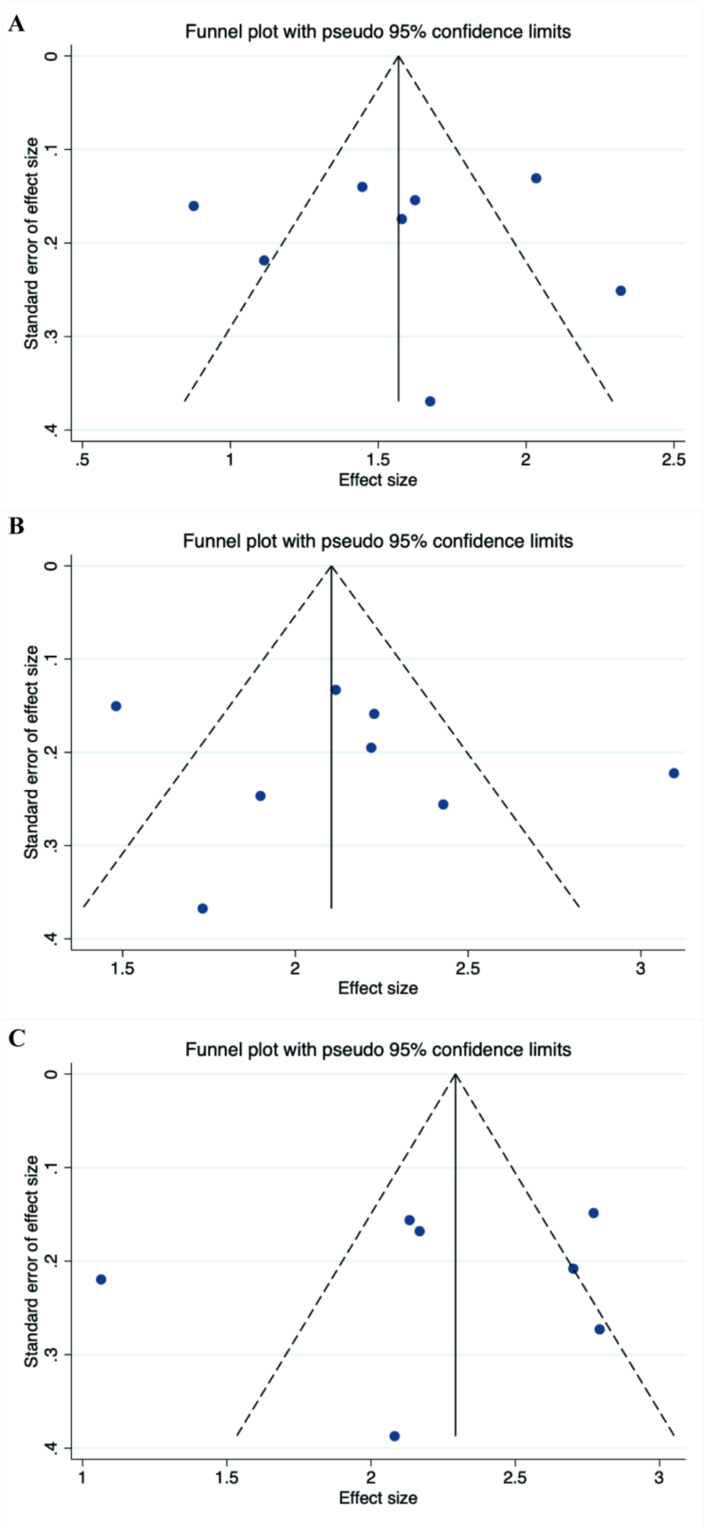

Supplemental Figure S8 Funnel plot of cord blood immunogenicity results

Note; (A) Anti-PT; (B) Anti-PRN; (C) Anti-FHA

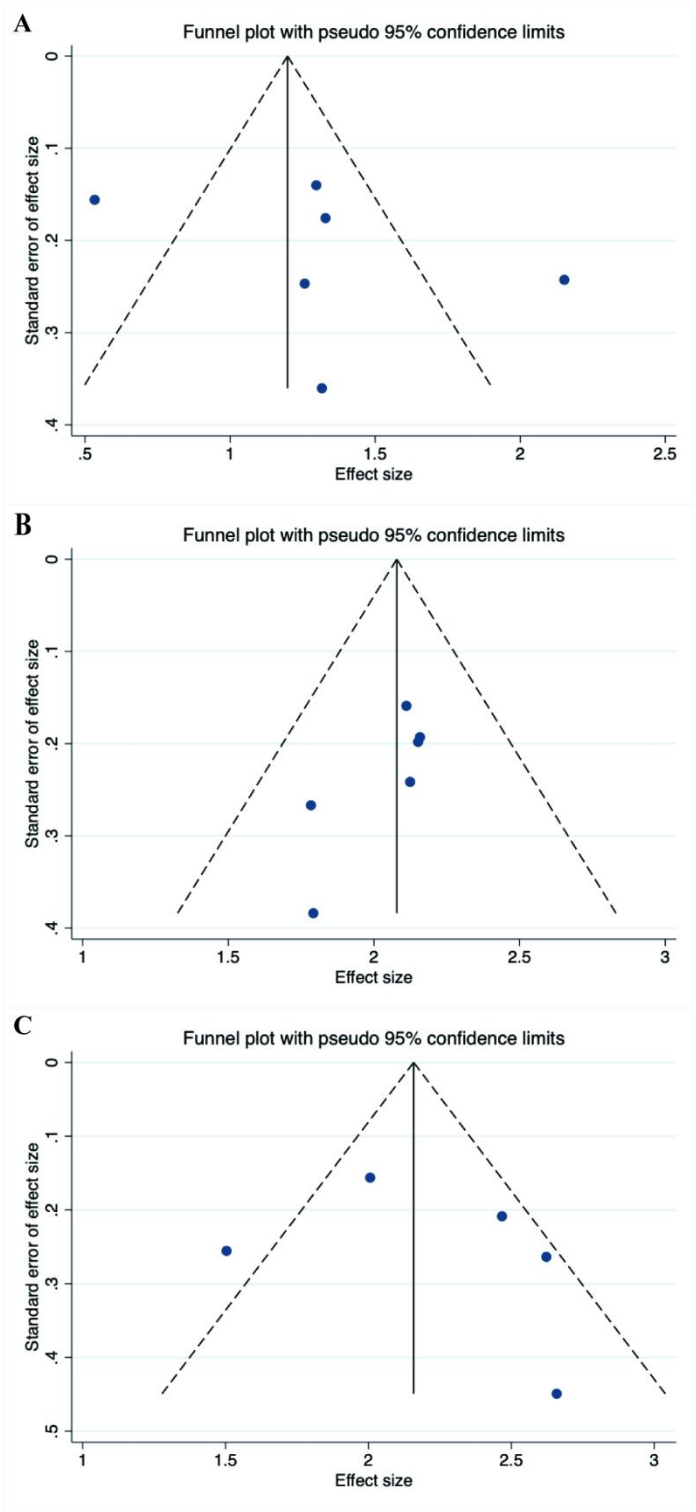

Supplemental Figure S9 Funnel plot of antibody levels before primary immunization in infants

Note; (A) Anti-PT; (B) Anti-PRN; (C) Anti-FHA

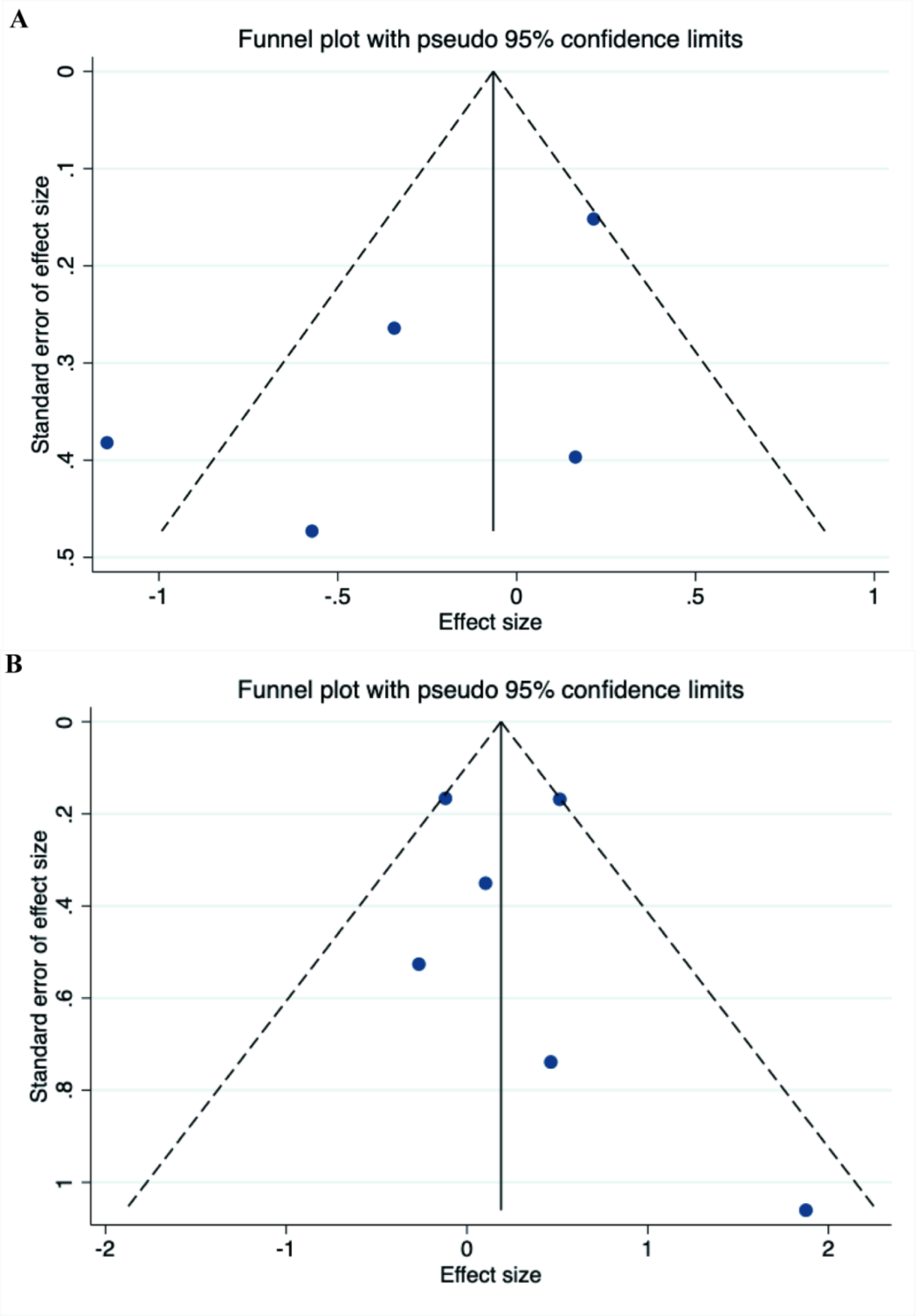

Supplemental Figure S10 Funnel plot on safety studies

Note: (A) infants; (B) pregnant woman
